# Enzymatic fine-tuning for 2-(6-hydroxynaphthyl) β-d-xylopyranoside synthesis catalyzed by the recombinant β-xylosidase BxTW1 from *Talaromyces amestolkiae*

**DOI:** 10.1186/s12934-016-0568-6

**Published:** 2016-10-04

**Authors:** Manuel Nieto-Domínguez, Alicia Prieto, Beatriz Fernández de Toro, Francisco Javier Cañada, Jorge Barriuso, Zach Armstrong, Stephen G. Withers, Laura I. de Eugenio, María Jesús Martínez

**Affiliations:** 1 Department of Environmental Biology, Centro de Investigaciones Biológicas, CSIC, Ramiro de Maeztu 9, 28040 Madrid, Spain; 2 Department of Chemical and Physical Biology, Centro de Investigaciones Biológicas, CSIC, Ramiro de Maeztu 9, 28040 Madrid, Spain; 3 Department of Chemistry, Centre for High-Throughput Biology, University of British Columbia, Vancouver, Canada

**Keywords:** β-Xylosidase, *Pichia pastoris*, N-Glycosylation, Transxylosylation, 2-(6-hydroxynaphthyl) β-d-xylopyranoside, Response surface methodology, Box-Behnken design

## Abstract

**Background:**

Glycosides are compounds displaying crucial biological roles and plenty of applications. Traditionally, these molecules have been chemically obtained, but its efficient production is limited by the lack of regio- and stereo-selectivity of the chemical synthesis. As an interesting alternative, glycosidases are able to catalyze the formation of glycosides in a process considered green and highly selective. In this study, we report the expression and characterization of a fungal β-xylosidase in *Pichia pastoris*. The transglycosylation potential of the enzyme was evaluated and its applicability in the synthesis of a selective anti-proliferative compound demonstrated.

**Results:**

The β-xylosidase BxTW1 from the ascomycete fungus *Talaromyces amestolkiae* was cloned and expressed in *Pichia pastoris* GS115. The yeast secreted 8 U/mL of β-xylosidase that was purified by a single step of cation-exchange chromatography. rBxTW1 in its active form is an N-glycosylated dimer of about 200 kDa. The enzyme was biochemically characterized displaying a *K*_*m*_ and *k*_*cat*_ against *p*-nitrophenyl-β-d-xylopyranoside of 0.20 mM and 69.3 s^−1^ respectively, and its maximal activity was achieved at pH 3 and 60 °C. The glycan component of rBxTW1 was also analyzed in order to interpret the observed loss of stability and maximum velocity when compared with the native enzyme. A rapid screening of aglycone specificity was performed, revealing a remarkable high number of potential transxylosylation acceptors for rBxTW1. Based on this analysis, the enzyme was successfully tested in the synthesis of 2-(6-hydroxynaphthyl) β-d-xylopyranoside, a well-known selective anti-proliferative compound, enzymatically obtained for the first time. The application of response surface methodology, following a Box-Behnken design, enhanced this production by eightfold, fitting the reaction conditions into a multiparametric model. The naphthyl derivative was purified and its identity confirmed by NMR.

**Conclusions:**

A β-xylosidase from *T. amestolkiae* was produced in *P. pastoris* and purified. The final yields were much higher than those attained for the native protein, although some loss of stability and maximum velocity was observed. rBxTW1 displayed remarkable acceptor versatility in transxylosylation, catalyzing the synthesis of a selective antiproliferative compound, 2-(6-hydroxynaphthyl) β-d-xylopyranoside. These results evidence the interest of rBxTW1 for transxylosylation of relevant products with biotechnological interest.

**Electronic supplementary material:**

The online version of this article (doi:10.1186/s12934-016-0568-6) contains supplementary material, which is available to authorized users.

## Background

β-Xylosidases (EC 3.2.1.37) together with endo-β-1,4-xylanases (EC 3.2.1.8) play a central role in the complete hydrolysis of xylans, the most abundant hemicelluloses in nature. During the past 20 years, β-xylosidases have been largely studied for their potential application in second-generation bioethanol production [1], as they improve the effectiveness of commercial enzymatic cocktails, usually with poor β-xylosidase activity [2]. However, these catalysts are interesting not only for their hydrolytic capacities, but also because many of these enzymes are able to catalyze transglycosylation reactions for glycoconjugate synthesis [3, 4].

**Fig. 1:**
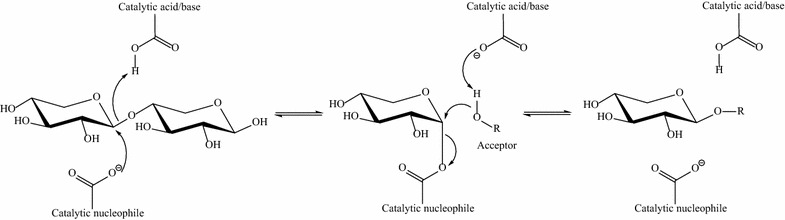
Transxylosylation reaction catalyzed by a retaining β-xylosidase

The class of enzymes that is useful in this regard are the retaining glycosidases, which catalyze hydrolysis with net retention of anomeric configuration. Such enzymes operate through a double-displacement mechanism involving the formation of a covalent glycosyl-enzyme intermediate, which is subsequently cleaved upon nucleophilic attack by water. Transglycosylation occurs when some other nucleophile than water takes part, giving rise to a new glycoside [5]. Transxylosylation is the particular name given to the reaction where a retaining β-xylosidase catalyzes the transfer of a xylose residue from a xylosyl donor, such as xylobiose, to an acceptor (Fig. 1).

The efficient synthesis of glycosides remains a significant challenge, especially for reactions that must be done on a large scale. Glycosides play important roles in biology. For example, the glycan moiety of glycoproteins and glycolipids is essential for many physiological processes, such as immune responses, cell adhesion or protein folding [5–7]. In addition, non-natural glycosides have been synthesized for a wide variety of applications, for example to enhance the properties of antioxidants or to generate new antibiotics [8, 9].

Chemical approaches to glycoconjugates synthesis typically require many protection and de-protection steps, in order to avoid the lack of regio- and stereo-selectivity displayed by chemical catalysts [10]. Currently, the use of glycosidases for synthesis of glycosides appears as one of the most interesting enzymatic alternatives. Under kinetically controlled conditions, these enzymes can glycosylate certain acceptors, with high selectivity and sensible yields, in a process considered green [3]. Unlike glycosyltransferases, which require high-cost nucleotide sugars as donors [10], glycosidases can use common and inexpensive glycans such as sucrose or starch, as sources of sugar donors [11, 12]. In the case of β-xylosidases, the use of xylobiose or other xylooligosaccharides (XOS), derived from the hydrolysis of xylan, may be a cost-effective alternative [13]. The attachment of a xylose moiety to a specific acceptor can result, for instance, in novel surfactants [14], antithrombotic drugs [15] or primers for studying the biosynthesis of heparan sulfate [16]. In fact, this priming role in the formation of glycosaminoglycans led to the development of specific xylosides as anti-proliferative compounds [17, 18], which have been successfully tested as selective inhibitors of the growth of tumor cells both in vitro and in vivo assays, as is the case for 2-(6-hydroxynaphthyl) β-d-xylopyranoside [19].

The purification and characterization of the BxTW1 β-xylosidase from *Talaromyces amestolkiae* has been recently reported. The enzyme, which belongs to the GH3 family (CAZy; http://www.cazy.org/), displays remarkable affinity and activity against xylobiose and XOS but, most importantly, it shows broad acceptor versatility and high regioselectivity in catalyzing transxylosylation reactions [20]. However, the amount of protein secreted by the native producer was quite low, which, when coupled with the poor purification yields reported severely limited its potential application. In this work, we report the expression of high levels of the recombinant β-xylosidase (rBxTW1) in the methylotrophic yeast *Pichia pastoris.* In addition, we describe the rBxTW1-catalyzed synthesis of 2-(6-hydroxynaphthyl) β-d-xylopyranoside using xylobiose as xylosyl donor and 2,6-dihydroxynaphthalene (2,6-DHN) as acceptor. To the best of our knowledge, this is the first report on enzymatic synthesis of this xyloside, which is known as a selective anti-proliferative compound [19]. The optimization of the reaction parameters by a Box-Behnken design (BBD) is also presented [21].

## Methods

### Enzyme and protein assays

β-Xylosidase activity was measured spectrophotometrically by the release of *p*-nitrophenol (*p*NP) (ε_410_ = 15,200 M^−1^ cm^−1^) from *p*-nitrophenyl β-d-xylopyranoside (*p*NPX) (Sigma-Aldrich). The standard assay was performed as described in Nieto-Domínguez et al. [20], but using sodium acetate buffer 50 mM (pH 5) instead of sodium citrate buffer. One unit of β-xylosidase activity was defined as the amount of enzyme that hydrolyzes 1 μmol of *p*NPX per minute in standard conditions.

The measurement of the β-xylosidase activity with xylobiose, xylooligosaccharides and beechwood xylan was performed by direct quantification of the released xylose. The d-xylose assay kit (Megazyme) was used for this purpose in accordance with the manufacturer’s instructions. In this case one unit of activity against xylobiose and XOS was defined as the amount of enzyme necessary for the complete hydrolysis of 1 μmol of the selected XOS to xylose per minute. Because of its polymeric nature, one unit of activity against xylan was considered to be the amount of enzyme necessary for releasing 1 μmol of xylose per minute.

Proteins were quantified by the bicinchoninic acid method by using the Pierce™ Protein Assay Kit (Thermo Scientific), according to the manufacturer’s instructions with bovine serum albumin as the standard.

### Isolation of fungal genomic DNA and cloning of the β-xylosidase gene

The selected *T. amestolkiae* strain is deposited in the IJFM (Instituto “Jaime Ferrán” de Microbiología) culture collection of the Centro de Investigaciones Biológicas, CSIC (Madrid, Spain) with the reference designation A795.

Genomic DNA was isolated from the fungus by using the DNeasy Plant Mini Kit in accordance with the manufacturer’s instructions. Primers were designed based on the nucleotide sequence of the *bxtw1* gene coding for BxTW1 from *T. amestolkiae* (GenBank accession no. KP119719), but excluding the region corresponding to the signal peptide, which was predicted by the SignalP 4.1 server (http://www.cbs.dtu.dk/services/SignalP/). Restriction sites for *EcoRI* and *NotI* were respectively added to the 5′ and 3′ primers (BxTw1 Fw, 5′-GAATTCCAGAACAACCAGACCTATGCCAATTACTCC-3′, and BxTw1 Rv, 5′-GCGGCCGCTTAATTGGGATCAGGTTGAATCTCCTGCTC-3′). The *bxtw1* gene was amplified by PCR using the genomic DNA as template. PCR mixtures contained 100 ng of DNA template, 1 μL PCR buffer, 1.5 mM MgCl_2_, 0.8 mM deoxynucleoside triphosphates, 0.5 μM each primer, and 1 U of *Expand™ High Fidelity PCR System* DNA polymerase (Sigma-Aldrich) in a final volume of 50 μL. Reaction mixtures were denatured at 94 °C for 5 min and then subjected to 34 cycles of amplification, each at 94 °C for 45 s, 55 °C for 45 s, and 72 °C for 2.5 min, followed by a final extension step at 72 °C for 7 min. Control reactions lacking DNA template were simultaneously performed.

The PCR product, encoding BxTW1 without its signal peptide, was ligated to the yeast expression vector pPIC9 (Invitrogen), fused with the α-factor signal sequence and expressed under the transcriptional control of the methanol-inducible AOX1 promoter. The pPIC9:bxtw1 construct was named pPICW1N and used for transforming *P. pastoris* KM71 and GS115 after linearization with *SacI* (New England Biolabs). Transformed colonies were grown on Yeast Nitrogen Base plates in the absence of histidine as selection marker.

### Screening for high-production clones

Transformed clones were screened for maximal production of recombinant BxTW1 (rBxTW1). The screening was performed on 25 clones from each strain. The colonies were cultured in a 96-well plate with 100 μL of YEPS medium (10 g/L yeast extract, 20 g/L peptone, 10 g/L sorbitol, 100 mM potassium phosphate buffer pH 6) per well. The medium included 5 g/L of methanol as inducer of gene expression. Cultures were incubated at 28 °C and 250 rpm and 50 μL of YEPS with 5 g/L of methanol were added at 24 and 48 h. Controls with both non-transformed *P. pastoris* KM71 and GS115 strains were included.

After 72 h the plate was centrifuged at 2000*g* for 15 min at 4 °C and 50 μL of each supernatant was placed into a new plate and incubated with 50 μL of a substrate solution containing 7 mM *p*NPX, 100 mM sodium formate buffer (pH 3) and 0.2 % BSA. The incubation was carried out at 50 °C for 10 min with gentle agitation. The reactions were stopped by the addition of 100 μL of 2 % Na_2_CO_3_ and the release of *p*NP was measured at 410 nm in a plate reader (SPECTRAMax Plus 384, Molecular Devices).

The two clones with highest activities from each strain were selected and liquid cultures were carried out as described below in order to compare clones in terms of secreted β-xylosidase activity.

### Production of rBxTW1

To prepare a fresh inoculum, the selected clones were grown overnight in 250-mL flasks with 50 mL of YEPS medium at 28 °C and 250 rpm. Then, rBxTW1 production was carried out in 1-L flasks with 100 mL of YEPS medium and 3.5 mL of the inoculum. Cultures were incubated at 28 °C and 250 rpm for 10 days with daily addition of 5 g/L methanol. Samples were periodically withdrawn as described above to measure β-xylosidase activity and absorbance at 600 nm (A_600_) as estimation of yeast growth.

### Purification of enzymes

For rBxTW1 purification, 10-day-old cultures were harvested and centrifuged at 10,000*g* and 4 °C for 20 min. The supernatant was sequentially filtered through 0.8-, 0.45- and 0.22-μm disc filters (Merck-Millipore). Then, the crude was first concentrated by tangential filtration and finally concentrated and dialyzed against 10 mM acetate buffer (pH 4) using a 50 kDa cutoff membrane (Merck-Millipore). rBxTW1 was purified by fast protein liquid chromatography (FPLC) using an ÄKTA Purifier chromatography system (GE Healthcare). The system was equilibrated in 10 mM sodium acetate buffer (pH 4) and the enzymatic crude was loaded onto a 5-mL Hi-Trap SPFF cartridge (GE Healthcare). The elution of the bound proteins was carried out by applying a linear gradient of 1 M NaCl from 0 to 50 % in 25 mL. The column was then washed with 1 M NaCl in 10 mL and re-equilibrated by applying 10 mL of the starting buffer. A flow rate of 1 mL/min was maintained during the entire process. Fractions with β-xylosidase activity were collected, pooled together, dialyzed and concentrated by ultrafiltration using 50 kDa cutoff Amicon Ultra-15 centrifugal devices (Merck-Millipore). The purified enzyme was stored at 4 °C.

The production of the native enzyme from *T. amestolkiae* and its further purification were carried out as described previously [20].

### Characterization of rBxTW1

#### Physicochemical properties

The enzyme was treated with endoglycosidase H (Endo H; Roche) in order to remove any N-glycosylation, according to manufacturer’s instructions. Purified enzymes, before and after N-deglycosylation, were analyzed by SDS-PAGE, mass spectrometry and size exclusion chromatography. Proteins were loaded onto 10 % polyacrylamide gels and stained with Coomassie brilliant blue R-250 (Sigma-Aldrich). Precision Plus protein dual color standards (Bio-Rad) were used in order to estimate its molecular mass. The accurate molecular mass and homogeneity of the pure enzyme were determined by matrix-assisted laser desorption ionization-time of flight mass spectrometry (MALDI-TOF) (Autoflex III, Bruker Daltonics) externally calibrated with Bovine Albumin from Sigma, covering the range from 30 to 150 kDa. The molecular mass in native conditions was studied by size-exclusion chromatography on a Superose 12 HR 10/30 column (GE Healthcare). Both MALDI-TOF and size-exclusion chromatography were performed as described previously [20].

The isoelectric point of the recombinant enzyme was analyzed by isoelectrofocusing as described by Salvachúa et al. [22] and β-xylosidase activity was detected as described by Yan et al. [23].

The effect of temperature and pH were studied in terms of stability and maximal activity. These assays were carried out by using 10 μg/mL of purified rBxTW1 and 0.1 % BSA, in order to get reproducible results regardless of the enzyme concentration [20].

The effect of pH was analyzed in a range from 2.2 to 7 for enzyme optimum activity and from 2.2 to 9 for enzyme stability. The selected buffers for each segment of the range were glycine–HCl (pH 2.2–3), sodium formate (pH 3–4), sodium acetate (pH 4–5.5), sodium histidine (5.5–7) sodium phosphate (pH 8) and Britton Robinson (pH 9) [24]. pH stability was determined by incubating the samples at 4 °C for 72 h, measuring β-xylosidase activity periodically in standard conditions.

The optimal temperature was analyzed by assaying β-xylosidase activity in 5 and 10 min reactions from 30 to 80 °C. Protein thermostability was described from its T50 value, a parameter defined as the temperature at which the enzyme loses 50 % activity after 10 min of incubation. The enzyme was incubated at 16 different temperatures along a range from 30 to 70 °C. Then, it was cooled at 4 °C for 10 min and rewarmed to room temperature for 5 min. The residual β-xylosidase activity was determined, taking the maximum measured value as 100 %.

#### Enzyme kinetics

Kinetics of rBxTW1 was evaluated against several synthetic and natural substrates for xylanolytic enzymes. The enzyme was assayed by using *p*NPX, *p*-NP-α-l-arabinopyranoside and *p*-NP-α-l-arabinofuranoside (Sigma-Aldrich). The analysis was also carried out against xylobiose, xylotriose, xylotetraose, xylopentaose and xylohexaose (Megazyme) together with beechwood xylan (Sigma-Aldrich).

Kinetics for each specific substrate were obtained by using increasing substrate concentrations from 0.078 to 20 mM. The activity data were fitted by least-squares to the Lineweaver–Burk linear equation of the Michaelis–Menten model. The effect of product inhibition from xylose was also determined by evaluating the *p*NPX hydrolysis in the presence of 2.5, 5 and 10 mM xylose and obtaining the corresponding *K*_*i*_.

#### Sugar analysis

To determine monosaccharide composition, protein samples were first hydrolyzed with 3 M trifluoroacetic acid (TFA, 121 °C, 1 h), and derivatized and analyzed as reported by Bernabé et al. [25]. The linkage types in the glycan chains of the protein were determined after methylation analysis of dry samples (1–3 mg), dissolved in dimethyl sulfoxide and processed according to the method of Ciucanu and Kerek [26]. The per-*O*-methylated polysaccharides were hydrolyzed with 3 M TFA, derivatized to their corresponding partially methylated alditol acetates, and analyzed by gas chromatography–mass spectrometry as described elsewhere [25].

### Specificity test of potential transxylosylation acceptors

A screening was carried out in order to evaluate the potential of several compounds to act as acceptors in transxylosylation reactions catalyzed by rBxTW1. The assay was performed in accordance with Blanchard and Withers [27] with minor variations. The enzyme was first inactivated as its 2-fluoroglucosyl species by incubating 150 µg/mL rBxTW1 in the presence of 5 mM 2,4-dinitrophenyl 2-deoxy-2-fluoro-β-d-xylopyranoside, 5 % dimethyl sulfoxide (DMSO), and 25 mM sodium phosphate (pH 6) in a final volume of 200 μL, at room temperature for 1 h. The sample was dialyzed by ultrafiltration with 10 kDa cutoff Viva spin 500 µL centrifugal filter units (Vivaproducts) in order to remove the excess of inhibitor. The final concentration of inactivated enzyme was corroborated by absorption at 280 nm. An aliquot of the purified, inactivated enzyme was added to the wells of a 96-well plate together with each compound to be screened and the buffer. The final reaction mix was composed of 2.9 μg/mL inactivated rBxTW1, 25 mM sodium phosphate buffer (pH 6), 0.1 % BSA and 20 mM or 40 % saturation of the potential acceptor. pH 6 was selected instead of the standard pH 5, in order to directly follow the release of *p*NP with time. Controls of non-inactivated enzyme and inactivated enzyme without any potential acceptor were included in triplicate.

The plate was incubated at room temperature for 1 h. Then, *p*NPX was added at a final concentration of 1 mM and the continuous change in absorbance of each well was measured at 400 nm and 40 °C for 1 h in a plate reader (Molecular Devices Spectra Max 190 Reader). Compounds leading to higher rate of recovery from inhibition than the non-acceptor control were considered positive hits and potential acceptors for transxylosylation by rBxTW1.

A library of 87 compounds was screened in order to find potential transxylosylation acceptors for rBxTW1. The assayed compounds were as follows: methanol; ethanol; 1-propanol; 2-propanol; 1-butanol; 1-pentanol; 1-hexanol; cyclohexanol; 1-octanol; 2-mercaptoethanol, 2-methoxyethanol; 3-mercapto-1-propanol; 5-hexyne-1-ol; 1-ethynylcyclohexanol; 1-adamantanemethanol; 1-pyrenemethanol; 1,2-ethanediol; 1,3-propanediol; phenol; phenethyl alcohol; *o*-phenylphenol; *p*-phenylphenol; 4-(hexyloxy)phenol; *p*-methoxyphenol; *p*-vinylphenol; resorcinol; phloroglucinol; gallic acid; caffeic acid; 1-naphtol; 2-naphtol; DL-threitol; l-erythritol; l-arabitol; d-arabitol; d-sorbitol; d-galactitol; d-mannitol; myo-inositol; phenyl β-d-glucopyranoside; phenyl β-d-galactopyranoside; *p*-nitrophenyl α-d-xylopyranoside; *p*-nitrophenyl α-l-arabinopyranoside; *p*-nitropheynl α-d-galactopyranoside; *p*-nitrophenyl α-d-mannopyranoside;; *p*-nitrophenyl β-d-glucopyranoside; *p*-nitropheynl β-d-galactopyranoside; *p*-nitrophenyl β-d-mannopyranoside; *p*-nitrophenyl β-d-fucopyranoside; *p*-nitrophenyl β-d-glucuronide; *p*-nitrophenyl β-d-cellobioside; *p*-nitrophenyl β-d-lactopyranoside; 4-methylumbilliferyl β-d-xylopyranoside; 4-methylumbelliferyl β-d-glucopyranoside; 4-methylumbelliferyl β-d-galactopyranoside; 4-methylumbelliferyl β-d-cellobiopyranoside; d-xylose; l-arabinose; d-lyxose; d-ribose; d-glucose; d-glucal; d-galactose; d-galactal; d-mannose; 1,5-anhydro-d-glucitol; d-tagatose; d-allose; l-sorbose; l-rhamnose; l-fucose; d-fructose; sucrose; d-maltose; α-lactose; α,α‐trehalose; d-cellobiose; gentiobiose; maltotriose; d-raffinose; l-serine; l-threonine; l-tyrosine; l-asparagine; l-arginine; l-cysteine. Stock solutions of these compounds were made in water to a final concentration, where possible, of 100 mM; some of these solutions were saturated.

### 2,6-Dihydroxynaphthalene as transxylosylation acceptor

The role of 2,6-DHN (Sigma-Aldrich) as a transxylosylation acceptor for rBxTW1 was assayed. The compound was added to the reaction at a final concentration of 3 g/L together with 0.01 g/L rBxTW1, 20 mM xylobiose, 0.1 % BSA, 50 mM sodium acetate buffer (pH 5) and 5 % acetonitrile. The reaction was incubated at 50 °C and 1200 rpm for 1 h. Samples were withdrawn at 10 min, 30 min and 1 h. The synthesis of new products was followed by thin layer chromatography (TLC) and high pressure liquid chromatography (HPLC).

TLCs were carried out by using silica gel G/UV254 polyester sheets, (0.2 mm thickness and 40 × 80 mm plate size) provided by Macherey–Nagel in ethyl acetate/methanol/water 10:2:1 (v/v). Detection was performed under 254 nm UV light by comparison of the pattern of spots between the reaction and a control without enzyme.

HPLC analyses were performed on an Agilent 1200 series LC instrument equipped with a ZORBAX Eclipse XDB-C18 column (Agilent). The system was equilibrated in acetonitrile/H_2_O 10:90 v/v (both containing 0.1 % acetic acid) with a flow of 2 mL/min. To purify the reaction products, acetonitrile concentration increased along a linear gradient from 10 to 20 % for 8 min. Then, the mobile phase mix changed to 95 % acetonitrile for 3 min and finally to 10 % for 3 min in order to respectively wash and re-equilibrate the column. The product peaks were detected by monitoring absorbance at 220 nm from the naphthalene ring and quantification was based on the areas under the peaks. As the reaction products are not commercially available, they were quantified from a calibration curve of 2,6-DHN.

### Response surface methodology

Optimization of the reaction conditions for the production of 2-(6-hydroxynaphthyl) β-d-xylopyranoside was attempted by using the response surface methodology. Design-Expert^®^ software version 10.0.1.0 (Stat-Ease Inc. MN, USA) was selected for generating a Box-Behnken design matrix and for the analysis of generated data. Concentration of xylobiose (donor), 2,6-DHN (acceptor) and enzyme, reaction time, temperature and pH were selected as the most significant parameters for xyloside production and included as independent variables for the development of the experimental design.

In this approach the parameters are studied at three levels: low, middle and high, leading to optimal values with a smaller number of designed experiments. The software then generates a polynomial quadratic equation from the obtained data which analyzes the effect of the independent variables on the response [28, 29].

The variables and levels assayed are displayed in Table 4. Maximum and minimum levels were previously determined by using one factor at a time approach (data not shown).

The reaction was scaled-up to 10 mL in conditions of high production predicted by the multiparametric model: 3 g/L 2,6-DHN, 50 mM xylobiose, 0.15 g/L rBxTW1 50 mM sodium acetate buffer (pH 5.5) and 39.5 °C for 80 min. The reaction was carried out at 1200 rpm and stopped by heating at 100 °C for 5 min.

The reaction mix was concentrated by speed vacuum before being loaded onto a semi-preparative column (Mediterranea sea_18_ TR-010006, Teknokroma) in order to be purified by HPLC. The system was equilibrated in acetonitrile/H_2_O 10:85 v/v (both containing 0.1 % acetic acid) and a flow rate of 2 mL/min was maintained during the entire process. The products were eluted by applying a gradient from 15 to 20 % acetonitrile for 13 min. Then the concentration of acetonitrile was raised up to 95 % for 3 min in order to wash the column. Finally, the system was allowed to re-equilibrate in the starting conditions for 4 min.

Product formation was followed by monitoring absorbance at 220 nm. After collection and solvent evaporation, products were stored at −20 °C.

### Nuclear magnetic resonance (NMR)

The identification of the xylosides formed from 2,6-dihydroxynaphthalene was carried out by NMR. Data were acquired at 308K, using a Bruker AVANCE 500 MHz spectrometer. 1D ^1^H NMR spectra, 2D homonuclear TOCSY (60 ms mixing time), and 2D heteronuclear ^1^H-^13^C HSQC experiments were acquired, in order to assign all NMR signals. For 1D ^1^H, TOCSY, and ^1^H-^13^C HSQC, the standard zg, zgesp, dipsi2phpr, and hsqcedetgp pulse sequences implemented in TOPSPIN 2.1 acquisition software (Bruker) were employed.

## Results and discussion

### Expression of rBxTW1 in *P. pastoris*

The gene *bxtw1* was successfully expressed in *P. pastoris*. The mature *bxtw1* sequence without signal peptide and introns comprised 2337 bp, including the native stop codon. After the screening, clone 18 from strain GS115 proved to be the best rBxTW1 producer and was selected for enzyme production in liquid cultures.

**Fig. 2:**
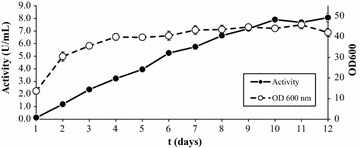
Extracellular β-xylosidase activity and absorbance at 600 nm of *P. pastoris* cultures in YEPS medium with 5 g/L methanol

β-Xylosidase activity reached a maximum of 8 U/mL in 10-day-old YEPS cultures (Fig. 2), which is an excellent value when compared with those reported for other fungal β-xylosidases of the GH3 family produced in *P. pastoris* (Table 1).

**Table 1:** Comparative production data of GH3 fungal β-xylosidases heterologously expressed in *P. pastoris*

Enzyme	Source	Production (U/mL)	References
rBxTW1	*Talaromyces amestolkiae*	8.0	This work
AnBX (Opt)	*Aspergillus niger ASKU28*	4.62	[45]
Xyl3A	*Humicola insolens*	1.16^a^	[46]
AnXln3D	*Aspergillus niger*	6.46	[47]
NCU09923	*Neurospora crassa*	3.04^a^	[48]
AN2359.2	*Aspergillus nidulans*	3	[49]
Bxl1	*Aureobasidium pullulans*	14.9	[50]
XylA	*Aspergillus japonicus*	0.33	[51]

^a^Not included in the original article but calculated with data provided

The recombinant rBxTW1 was completely purified by FPLC after a single step of cation-exchange chromatography with a yield of 91.5 % and a degree of purification of 1.9. The above data represent a huge increase in both maximal activity and protein purification yield respect to the native enzyme, which is produced at 0.8 U/mL and purified after three chromatographic steps, with a final yield of 10.8 % [20]. These results indicate that *P. pastoris* is an appropriate host for producing rBxTW1.

### Characterization of rBxTW1

**Fig. 3:**
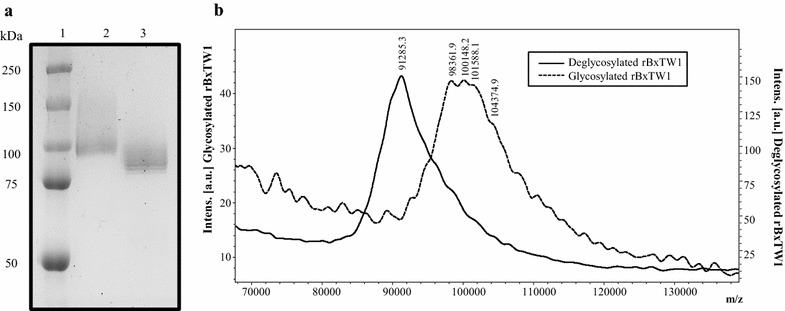
Estimation of rBxTW1 molecular mass by **a** SDS-PAGE and **b** MALDI-TOF MS. *Lanes 1* molecular mass standards; *2* glycosylated BxTW1; *3* BxTW1 treated with Endo H. Intens., intensity; a.u., arbitrary units

The isoelectric point of the recombinant enzyme was determined to be 8.4, more basic than that of the native enzyme, which is probably due to their different glycosylation pattern [30]. The molecular mass estimated by SDS-PAGE was around 100 kDa. The difference between this value and that predicted from the amino acid sequence of the protein (84.650 kDa) suggested that rBxTW1 was glycosylated, which is common for proteins expressed in *P. pastoris* [31]. The enzyme was N-deglycosylated with Endo H and subsequently analyzed by SDS-PAGE and MALDI-TOF MS (Fig. 3). As expected, the molecular mass decreased, displaying a value of 91 kDa by MALDI-TOF. The difference with respect to the theoretical 84.650 kDa may be attributed to O-glycosylation which are also introduced by the yeast but not removed with Endo H. These assays confirmed that *P. pastoris* was producing the enzyme as a glycoprotein with approximately 10 % N-glycosylation. On the other hand, the measured molecular mass of rBxTW1 determined by size exclusion chromatography was around 160 kDa, suggesting that in aqueous medium this protein forms a non-covalent dimer, as reported for the native enzyme [20]. As the values obtained by size exclusion chromatography are influenced by the shape of the proteins, and are not fully accurate, hereinafter the correct average mass of the glycosylated monomeric rBxTW1 will be assumed to be 100 kDa, as determined by SDS-PAGE, and that of the dimeric form 200 kDa. This molecular mass value is the one used to calculate the kinetic parameters of the β-xylosidase.

The kinetic properties of rBxTW1 were determined against *p*NP-pentoses, XOS and beechwood xylan (Table 2). The enzyme showed high affinity for *p*NPX and XOS, especially when compared with the increased *K*_*m*_ displayed in the hydrolysis of *p*NP-α-l-arabinopyranoside and *p*NP-α-l-arabinofuranoside. Competitive inhibition by product (xylose) was analyzed resulting in a *K*_i_ of 1.7 ± 0.3 mM when *p*NPX was used as substrate.

**Table 2:** Kinetic parameters of rBxTW1

Substrate	*K*_*m*_ (mM)	*V*_*max*_ (U/mg)	*k*_*cat*_ (s^−1^)	*k*_*cat*_/*K*_*m*_ (mM^−1^ s^−1^)
*p*NPX	0.20 ± 0.01	20.8 ± 0.3	69.3	336
*p*NP-α-l-arabinopyranoside	1.6 ± 0.1	8.3 ± 0.2	28	17
*p*NP-α-l-arabinofuranoside	4.3 ± 0.3	14.9 ± 0.4	49.8	12
Xylobiose	0.51 ± 0.03	26.4 ± 0.3	87.9	170
Xylotriose	0.19 ± 0.01	9.0 ± 0.1	30	160
Xylotetraose	0.20 ± 0.01	6.34 ± 0.06	21.1	100
Xylopentaose	0.22 ± 0.01	4.48 ± 0.05	14.9	68
Xylohexaose	0.21 ± 0.02	4.27 ± 0.07	14.2	69

Polymeric substrate	*K*_*m*_ (mg/mL)	*V*_*max*_ (U/mg)	*k*_*cat*_ (s^−1^)	*k*_*cat*_/*K*_*m*_ (mM^−1^ s^−1^)
Xylan	8.9 ± 0.3	21.4 ± 0.3	71.5	–

**Fig. 4:**
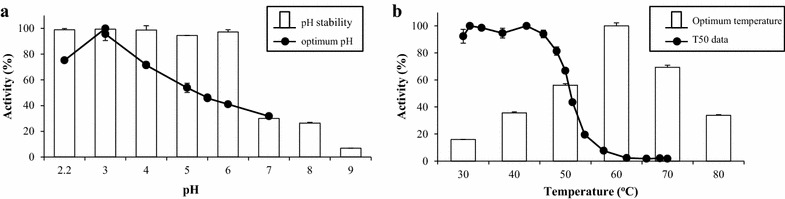
Effect on rBxTW1 activity of: **a** pH and **b** temperature. **a** The *line* indicates the effect of pH on enzyme activity, and the *bars* show its stability over a range of pH values from 2.2 to 9 after 72 h. **b** The *line* displays the evolution of residual activity for T50 determination, and the *bars* correspond to the effect of the reaction temperature on enzyme activity

The biochemical and kinetic properties of rBxTW1, namely the general profiles for optimal pH (Fig. 4a) and temperature (Fig. 4b), substrate specificity, and high affinity for *p*NPX and XOS, were very similar to those reported for the native enzyme [20]. However, the temperature for maximal activity decreased from 70 to 60 °C, T50 was around 9° lower and the enzyme lost its stability at basic pH, indicating the lower stability of the recombinant form. In addition, a decrease of its maximum velocity was also observed. Similar results have been reported by Wei et al. expressing a fungal GH3 β-glucosidase in *P. pastoris* [32], which were attributed to the fact that N-glycans usually introduced by this yeast are larger than those of fungi.

The native and recombinant BxTW1 enzymes both carry glycosylation at approx. 10 % of their mass [20]. However, the compositions of their glycans are different (Table 3). Both enzymes had mannose as the major monosaccharide, but the carbohydrate chains of the β-xylosidase secreted by *T. amestolkiae* also contained substantial amounts of glucose and N-acetyl-glucosamine, while in rBxTW1 mannose residues represented more than 90 % of its total sugar content, which is consistent with the hyper-mannosylation described for yeasts [33]. In addition, these enzymes also differed in the linkage types of their glycan component (Table 3). The interpretation of the data will be focused on the mannose content, which represents the main part of the glycan and has been deeply studied in the N-glycosylation patterns reported for fungi and yeasts [34, 35]. Mannose residues with a single link assigned (Man*p*-(1→) represent the non-reducing ends of oligosaccharide chains, two links meant a residue for chain extension (→2)-Man*p*-(1→; →3)-Man*p*-(1→) while three or four links implied a branching point. Based on this, there are important differences between the recombinant and the native fungal enzyme. rBxTW1 displays about 35 % of →2)-Man*p*-(1→ units and low content of the other types of extension-residues, while in the native enzyme the proportion of →2)-Man*p*-(1→ and →6)-Man*p*-(1→ units is similar (18 and 16 % respectively). On the other hand, the native fungal enzyme was found to have 6 % branching points for triple branching, whereas these residues represent less than 1 % in rBxTW1. These data suggest that the glycan in the enzyme from *T. amestolkiae* has a highly branched structure, but with shorter chains in comparison with the recombinant enzyme, which also displayed more homogeneity.

**Table 3:** Monosaccharide distribution and linkage types present in the carbohydrate moiety of the native and recombinant BxTW1

	Characteristic ions (m/z)	Content (%)	
		Native	Recombinant
Monosaccharide			
Mannose	43, 115, 145, 187, 217, 259, 361	68.7	91.9
Glucose	43, 115, 145, 187, 217, 259, 361	13.1	1.9
Glucosamine	43, 84, 102, 144, 156, 258, 318, 360	12.1	6.2
Galactose	43, 115, 145, 187, 217, 259, 361	6.1	–
Deduced linkage			
Man*p*-(1→	87, 88, 102, 118, 129, 161, 205	27.5	29.0
Gal*p*-(1→	89, 101, 102, 118, 162, 205	3.0	0.4
→2)-Man*p*-(1→	87, 88, 101, 129, 130, 161, 190	18.1	35.3
→3)-Man*p*-(1→	101, 118, 129, 161, 234	0.0	2.0
→6)-Man*p*-(1→	87, 88, 99, 102, 118, 129, 162, 189	16.4	3.4
→2,3)-Man*p*-(1→	101, 129, 161, 202, 262	0.0	1.3
→2,6)-Man*p*-(1→	117, 118, 129, 130, 189, 190	1.9	14.0
→3,6)-Man*p*-(1→	118, 129, 189, 174, 234	14.5	8.1
→3,4,6)-Man*p*-(1→	118, 139	6.3	0.5
→4)-Glc*p*NH_2_-(1→	117, 159, 233	12.3	4.6

The differences in carbohydrate content and structure between the two enzymes are in good accordance with the hypothesis proposed by Wei et al. [32], suggesting that the long mannose-chains incorporated by *P. pastoris*, in opposition to the shorter and more heterogeneous fungal glycosylation, may be the primary cause of the differences between the two glycosidases.

However, the drawbacks observed for the recombinant enzyme are compensated by its high production levels and purification yields, since expression in *P. pastoris* led to an increase of 85-fold on the recovered activity units per volume of culture.

### rBxTW1 as a versatile tool for transxylosylation of bioactive compounds

In order to fully determine the biotechnological potential of the recombinant enzyme, its transxylosylation capacities were analyzed. To test if rBxTW1 kept the transglycosylation potential exhibited by the native enzyme [20], and in particular to assess its aglycone preference, a rapid screen of aglycone specificity was performed, according to the methodology developed by Blanchard and Withers [27]. The library of tested compounds included confirmed acceptors for the native enzyme, representative carbohydrates, alcohols and aromatic compounds together with some amino acids and other chemicals readily available in the laboratory.

**Fig. 5:**
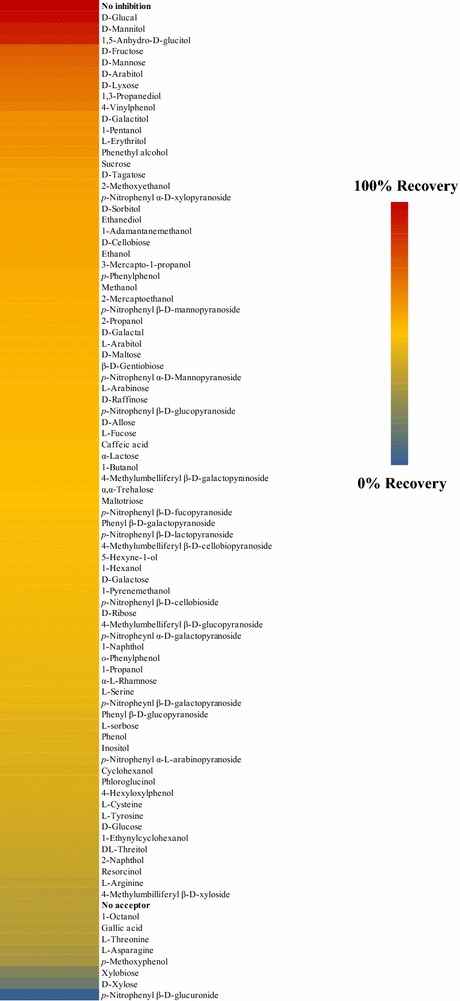
Heat map of inhibition recovery of rBxTW1 in the presence of the assayed compounds. Those compounds giving values between no-acceptor and the no-inhibition controls were considered potential acceptors of transxylosylation

The results from this study are gathered as a heat map at Fig. 5. The high number of positive hits suggested that the recombinant enzyme retained the broad acceptor versatility reported for BxTW1. Sugar alcohols seemed to be very good acceptors for both enzymes. The presence of gallic acid and *p*-nitrophenyl β-d-glucuronide among the few negative hits suggested that carboxylic acids are not suitable acceptors for being transxylosylated by rBxTW1. Xylobiose and xylose also appeared as apparent negative hits, which is at first sight surprising because both carbohydrates are well known acceptors for BxTW1 ([20]; Additional file 1). This is due to the fact that xylose and xylobiose are, respectively, a competitive inhibitor and a substrate for rBxTW1, thus competed with *p*NPX in the assay, thereby appearing not to be acceptors. The same could be true for *p*-nitrophenyl β-d-glucuronide. This emphasizes the need to carry out a parallel screen with non-inactivated enzyme to check for potential inhibitors of this class, so that they can be investigated separately.

Among the clearest positive hits there were at least three remarkable compounds for their potential biotechnological importance in terms of transglycosylation. 4-vinylphenol is a volatile phenolic compound found in wine, beer and other non-alcoholic beverages as orange juice. It is released from precursors during storage, being one of the main causes of off-flavor problems. Transxylosylation may provide a solution to this industrial concern by converting this molecule into a stable non-volatile glycoside [36, 37]. The second interesting compound is 1-pentanol that can be transformed into pentyl xyloside, an alkyl xyloside that, like others, may be a useful surfactant [38]. Regarding phenethyl alcohol, its antimicrobial properties are well known and natural glycosides of this compound have been found and reported to display immunosuppressive responses [39, 40]. The chemical synthesis of glycosides from 4-vinylphenol, 1-pentanol and phenethyl alcohol needs a minimum of three steps Therefore, the easily accessible synthesis of those xylosides and related derivatives by transxylosylation and the study of their potential biological activity may lead to a better understanding of these properties. To the best of our knowledge, there is only one precedent of using phenethyl alcohol [41] and no previous report concerning the use of 4-vinylphenol as acceptors for enzymatic transglycosylation.

### Enzymatic synthesis of 2-(6-hydroxynaphthyl) β-d-xylopyranoside and production enhancement by response surface methodology

The screening of the aglycone specificity also revealed 2-naphthol and especially 1-naphthol as potential transxylosylation acceptors for rBxTW1. This was an unexpected result since the bulky naphthalene molecule might not have been expected to fit in enzyme active site. These data prompted us to test for the formation of a new xyloside by using 2,6-DHN as an acceptor, due to the existing structural similarities between this compound and 1- and 2-naphtol, and because the potential transxylosylation product would be 2-(6-hydroxynaphthyl) β-d-xylopyranoside, a well known antiproliferative compound. The three compounds contain a naphthalene ring which is monohydroxylated in the case of 1- and 2-naphtol and dihidroxylated for 2,6-DHN. The absence of any other significant difference led us to expect that this compound would also work as transxylosylation acceptor for rBxTW1. The desired xyloside, 2-(6-hydroxynaphthyl) β-d-xylopyranoside, functions as selective inhibitor of transformed or tumor-derived cell growth by acting as an alternative primer for heparan sulfate synthesis. These interesting properties had been widely demonstrated in vitro by adding the compound to cultures of normal and transformed cell lines such as HFL-1 cells (human fetal lung fibroblasts), 3T3 A31 cells (mouse 3T3 fibroblasts) and T24 cells (human bladder carcinoma cells) and in vivo with a subcutaneous tumor model in mice [19, 42].

Since the selected library did not include 2,6-DHN, the role of this compound as acceptor for rBxTW1 was evaluated by the direct detection of the new product in TLC, which is a better approach when assaying single compounds. With the set of conditions initially fixed for the transxylosylation assay, a faint new spot, probably corresponding to the reaction product, was observed upon developing the TLC plate (data not shown). HPLC analysis of the reaction mixture allowed estimation of the concentration of the hypothetical new xyloside to be 0.19 mM. The reaction conditions were further optimized using a response surface method, specifically BBD, in order to enhance the production of 2-(6-hydroxynaphthyl) β-d-xylopyranoside. The matrix of the experiments generated by the BBD approach and the outcomes from this analysis are collected in Table 4. The BBD matrix with the production data was analyzed by Design-Expert^®^ software and fitted to the following quadratic model equation:

**Table 4:** Box–Behnken experimental design for optimization of 2-(6-hydroxynaphthyl)-β-d-xylopyranoside

2,6-DHN (g/L)	Temperature (°C)	Xylobiose (mM)	Time (min)	Enzyme (g/L)	pH	[Product] (mM)
0.30	40	60	10	0.055	4.1	0.04
3.00	40	60	10	0.055	4.1	0.45
0.30	60	60	10	0.055	4.1	0.05
3.00	60	60	10	0.055	4.1	0.37
0.30	40	60	60	0.055	4.1	0.13
3.00	40	60	60	0.055	4.1	1.09
0.30	60	60	60	0.055	4.1	0.12
3.00	60	60	60	0.055	4.1	0.55
1.65	40	20	35	0.010	4.1	0.13
1.65	60	20	35	0.010	4.1	0.05
1.65	40	100	35	0.010	4.1	0.13
1.65	60	100	35	0.010	4.1	0.07
1.65	40	20	35	0.100	4.1	0.34
1.65	60	20	35	0.100	4.1	0.41
1.65	40	100	35	0.100	4.1	0.80
1.65	60	100	35	0.100	4.1	0.83
1.65	50	20	10	0.055	2.2	0.23
1.65	50	100	10	0.055	2.2	0.11
1.65	50	20	60	0.055	2.2	0.37
1.65	50	100	60	0.055	2.2	0.55
1.65	50	20	10	0.055	6.0	0.26
1.65	50	100	10	0.055	6.0	0.29
1.65	50	20	60	0.055	6.0	0.48
1.65	50	100	60	0.055	6.0	0.80
0.30	50	60	10	0.010	4.1	0.00
3.00	50	60	10	0.010	4.1	0.07
0.30	50	60	60	0.010	4.1	0.05
3.00	50	60	60	0.010	4.1	0.31
0.30	50	60	10	0.100	4.1	0.09
3.00	50	60	10	0.100	4.1	0.85
0.30	50	60	60	0.100	4.1	0.11
3.00	50	60	60	0.100	4.1	1.31
1.65	40	60	35	0.010	2.2	0.18
1.65	60	60	35	0.010	2.2	0.00
1.65	40	60	35	0.100	2.2	0.67
1.65	60	60	35	0.100	2.2	0.19
1.65	40	60	35	0.010	6.0	0.13
1.65	60	60	35	0.010	6.0	0.02
1.65	40	60	35	0.100	6.0	0.77
1.65	60	60	35	0.100	6.0	0.49
0.30	50	20	35	0.055	2.2	0.05
3.00	50	20	35	0.055	2.2	0.49
0.30	50	100	35	0.055	2.2	0.10
3.00	50	100	35	0.055	2.2	0.66
0.30	50	20	35	0.055	6.0	0.06
3.00	50	20	35	0.055	6.0	0.66
0.30	50	100	35	0.055	6.0	0.11
3.00	50	100	35	0.055	6.0	0.67
1.65	50	60	35	0.055	4.1	0.68
1.65	50	60	35	0.055	4.1	0.69
1.65	50	60	35	0.055	4.1	0.67
1.65	50	60	35	0.055	4.1	0.67
1.65	50	60	35	0.055	4.1	0.70
1.65	50	60	35	0.055	4.1	0.65
1.65	50	60	35	0.055	4.1	0.67
1.65	50	60	35	0.055	4.1	0.66
1.65	50	60	35	0.055	4.1	0.66
1.65	50	60	35	0.055	4.1	0.68
1.65	50	60	35	0.055	4.1	0.66

[Product] = − 3.82 + 0.395A + 0.128B + 1.41 × 10^−3^C + 1.51 × 10^−2^D + 1.12E + 0.144F − 5.69 × 10^−3^AB + 2.06 × 10^−4^AC + 2.38 × 10^−3^AD + 3.32AE + 7.58 × 10^−3^AF − 5.46 × 10^−6^BC − 2.36 × 10^−4^BD −2.88 × 10^−2^BE + 1.77 × 10^−3^BF + 7.48 × 10^−5^CD + 5.99 × 10^−2^CE + 1.13 × 10^−4^CF + 1.90 × 10^−2^DE + 3.72 × 10^−4^DF + 0.629EF − 6.54 × 10^−2^A^2^ − 1.23 × 10^−3^B^2^ − 5.16 × 10^−5^C^2^ − 1.31 × 10^−4^D^2^ − 60.8E^2^ −3.34 × 10^−2^F^2^

A: [2,6-DHN] (g/L); B: Temperature (°C); C: [Xylobiose] (mM); D: Time (min); E: [Enzyme] (g/L); F: pH.

The model predicted the production of the new xyloside as a function of the concentrations of 2,6-DHN and xylobiose, the amount of added enzyme, the temperature and time of reaction and the pH value. The analysis of variance test calculated by the software (Table 5) validated that the model matched the experimental data.

**Table 5:** ANOVA report from the quadratic model for xyloside production

Source	Sum of squares	df	Mean square	F value	p value prob > F^a^
Model	5.59	27	0.21	32.28	<0.0001
Residual	0.21	32	6.42 × 10^−3^		
Lack of fit	0.20	21	9.67 × 10^−3^	48.72	<0.0001
Pure error	2.18 × 10^−3^	11	1.99 × 10^−4^		
Cor total	5.59	27	0.21	32.28	<0.0001

^a^Values of prob > F less than 0.0500 indicate model terms are significant

The Design-Expert^®^ software was used to find the experimental conditions for maximal production predicted by the model within the selected limits for each parameter. The software generated a maximum value of 1.6 mM, which required a reaction mix composed of 3 g/L 2,6-DHN; 100 mM xylobiose; 0.1 g/L rBxTW1 and 50 mM sodium acetate pH 4.8, a temperature of 40.8 °C and a reaction time of 60 min. Under these conditions, the measured product concentration fit the theoretical value of 1.6 mM. Therefore, application of the response surface model enabled an eightfold increase (from 0.19 to 1.6 mM) in xyloside production.

The reaction parameters from the former Box-Behnken adjustment were further changed according to the availability of reactants and enzyme in order to produce the hypothetical product 2-(6-hydroxynaphthyl) β-d-xylopyranoside for its purification and identification. Since pure commercial xylobiose is an expensive substrate, but the enzyme is easily produced and purified, a new estimation was run by the model, decreasing xylobiose concentration from 100 to 50 mM, while no limits were set on enzyme concentration, temperature and reaction time. With these new settings, the predicted optimal conditions included higher enzyme concentration and longer reaction time (0.1 to 0.15 g/L and 60 to 80 min, respectively), while temperature was slightly lower to delay enzyme inactivation and pH changed from 4.8 to 5.5. Under these conditions, a maximum concentration of 1.5 mM was predicted, and an actual value of 1.4 mM was obtained in this assay. This value is close to the 1.6 mM achieved when no limits were applied to xylobiose concentration. In both cases, the empirical values corroborated the theoretical predicted data.

The reaction mix was concentrated and analyzed by semi-preparative HPLC, purifying a major product peak and a minor one (Product 2) that was not previously detected in analytical-scale reactions. The yield of this by-product was, however, very low (0.2 mM). Product 2 eluted during the acetonitrile gradient, before the hypothetical 2-(6-hydroxynaphthyl) β-d-xylopyranoside (Product 1), suggesting that Product 2 may have incorporated a second xylose unit and thereby acquired increased polarity. If this assumption is true, the most probable scenarios are a second xylose unit attached either to the remaining free hydroxyl group of the 2-(6-hydroxynaphthyl) β-d-xylopyranoside or to the xylose already present. As the demonstrated regioselectivity of the native enzyme [20] is expected to persist in the recombinant form, the latter option may occur through a β(1 → 4) linkage, converting the xylose substituent into xylobiose. For complete identification of products, they were analyzed by NMR as follows.

### Structural elucidation of the 2,6-dihydroxynaphthyl transxylosylation products by NMR

**Fig. 6:**
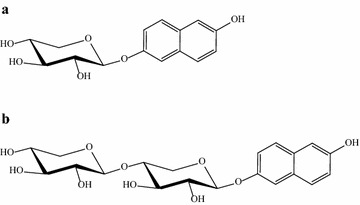
**a** 2-(6-hydroxynaphthyl) β-d-xylopyranoside (product 1) and **b** 2-(6-hydroxynaphthyl) β-d-xylobioside (product 2) synthesized by rBxTW1 catalyzed transxylosylation. **a** Product 1 is formed in one step when a xylose moiety is attached to an hydroxyl group of 2,6-DHN. **b** The attachment of a second xylose to the former one by a β(1 → 4) linkage converts product 1 into product 2

Analysis of ^1^H and ^13^C-NMR experiments of the two purified products allowed the elucidation of their structure. As expected, Product 1 was found to be 2-(6-hydroxynaphthyl) β-d-xylopyranoside (Fig. 6a; Table 6) in accordance with data described for the chemically synthesized compound [43] while product 2, where two different sets of xylose signals appeared, was identified as 2-(6-hydroxynaphthyl) β-d-xylobioside (Fig. 6b; Table 6). The synthesis of the latter product instead of 2,6-dihydroxynaphthalene bis(β-d-xylopyranoside), that due to symmetry of the molecule would have only one set of xylose signals [44], indicated the preference of the enzyme for the sugar hydroxyl over the second naphthyl alcohol.

**Table 6:** Chemical shift data from 2-(6-hydroxynaphthyl) β-d-xylopyranoside and 2-(6-hydroxynaphthyl) β-d-xylobioside

	2-(6-hydroxynaphthyl) β-d-xylopyranoside		2-(6-hydroxynaphthyl) β-d-xylobioside	
	^1^H (ppm)	^13^C (ppm)	^1^H (ppm)	^13^C (ppm)
H1′	–	–	4.54	102.14
H2′			3.33	73.05
H3′			3.49	75.88
H4′			3.69	69.50
H5′			3.37	65.50
H5′			4.04	
H1	5.22	101.35	5.24	101.27
H2	3.65	73.17	3.69	72.98
H3	3.64	75.79	3.76	73.95
H4	3.78	69.39	3.94	76.60
H5	3.56	65.50	3.64	63.13
H5	4.10		4.23	
Naphthalene ring	7.24	119.21	7.24	119.21
	7.31	109.64	7.31	109.64
	7.34	119.59	7.34	119.59
	7.53	111.78	7.53	111.78
	7.82	128.49	7.82	128.49
	7.84	129.27	7.84	129.27

As far as we know the synthesis of 2-(6-hydroxynaphthyl) β-d-xylobioside has not been reported previously. The chemical synthesis of this compound would be more challenging than that of the xyloside because of the need to attach the second xylose moiety specifically to the C-4 hydroxyl group of the acceptor sugar [10]. The potential anti-proliferative role of 2-(6-hydroxynaphthyl)-β-d-xylobioside has not yet been investigated and merits attention. Further research using new and cheaper xylose donor will be necessary in order to carry out the required increase of production for the anti-proliferative analyses.

## Conclusions

A β-xylosidase from *T. amestolkiae* was produced in *P. pastoris* and purified. The final yields were much higher than those attained for the native protein, although some loss of stability and maximum velocity was observed. rBxTW1 displayed remarkable acceptor versatility in transxylosylation, catalyzing the synthesis of a selective antiproliferative compound, 2-(6-hydroxynaphthyl) β-d-xylopyranoside, enzymatically obtained for the first time. Response surface approaches enhanced this production by eightfold. These results evidence the interest of rBxTW1 for transxylosylation of industrially relevant products. Using xylans from lignocellulosic wastes, as cost-effective xylose donors, should be tested for developing a green alternative to current chemical synthesis.

## Additional file

10.1186/s12934-016-0568-6 Xylose and xylobiose as transxylosylation acceptors. Transxylosylation reactions catalyzed by rBxTW1 using xylose or xylobiose as acceptors. The analysis by TLC of the samples is displayed in order to demonstrate the synthesis of the transxylosylation products.

## Abbreviations

2-ME: 2-mercaptoethanol
A_600_: absorbance at 600 nm
BBD: Box-Behnken design
2,6-DHN: 2,6-dihydroxynaphthalene
DMSO: dimethyl sulfoxide
DTT: dithiothreitol
FPLC: fast protein liquid chromatography
MALDI-TOF: matrix-assisted laser desorption ionization-time of flight mass spectrometry
NMR: nuclear magnetic resonance
*p*NP: *p*-nitrophenol
*p*NPX: *p*-nitrophenyl β-d-xylopyranoside
rBxTW1: recombinant BxTW1
TFA: trifluoroacetic acid
TLC: thin layer chromatography
XOS: xylooligosaccharides

## References

[1] Knob A, Terrasan C, Carmona E (2010). β-xylosidases from filamentous fungi: an overview. World J Microbiol Biotechnol.

[2] Bao L, Huang Q, Chang L, Sun Q, Zhou J, Lu H (2012). Cloning and characterization of two β-glucosidase/xylosidase enzymes from yak rumen metagenome. Appl Biochem Biotechnol.

[3] Hermida C, Corrales G, Canada FJ, Aragon JJ, Fernandez-Mayoralas A (2007). Optimizing the enzymatic synthesis of beta-d-galactopyranosyl-d-xyloses for their use in the evaluation of lactase activity in vivo. Bioorg Med Chem.

[4] Kim DY, Ham SJ, Kim HJ, Kim J, Lee MH, Cho HY, Shin DH, Rhee YH, Son KH, Park HY (2012). Novel modular endo-beta-1,4-xylanase with transglycosylation activity from *Cellulosimicrobium* sp strain HY-13 that is homologous to inverting GH family 6 enzymes. Bioresour Technol.

[5] Wang LX, Huang W (2009). Enzymatic transglycosylation for glycoconjugate synthesis. Curr Opin Chem Biol.

[6] Shental-Bechor D, Levy Y (2009). Folding of glycoproteins: toward understanding the biophysics of the glycosylation code. Curr Opin Struct Biol.

[7] Mattner J, Debord KL, Ismail N, Goff RD, Cantu C, Zhou DP, Saint-Mezard P, Wang V, Gao Y, Yin N, Hoebe K, Schneewind O, Walker D, Beutler B, Teyton L, Savage PB, Bendelac A (2006). Exogenous and endogenous glycolipid antigens activate NKT cells during microbial infections. Nature.

[8] Regev-Shoshani G, Shoseyov O, Bilkis I, Kerem Z (2003). Glycosylation of resveratrol protects it from enzymic oxidation. Biochem J.

[9] Baltz RH (2006). Molecular engineering approaches to peptide, polyketide and other antibiotics. Nat Biotecnol.

[10] Danby PM, Withers SG (2016). Advances in enzymatic glycoside synthesis. ACS Chem Biol..

[11] Torres P, Poveda A, Jimenez-Barbero J, Luis Parra J, Comelles F, Ballesteros AO, Plou FJ (2011). Enzymatic synthesis of alpha-glucosides of resveratrol with surfactant activity. Adv Synth Catal.

[12] Lu L, Fu F, Zhao R, Jin L, He C, Xu L, Xiao M (2014). A recombinant levansucrase from *Bacillus licheniformis* 8-37-0-1 catalyzes versatile transfructosylation reactions. Process Biochem.

[13] Jain I, Kumar V, Satyanarayana T (2014). Applicability of recombinant β-xylosidase from the extremely thermophilic bacterium *Geobacillus thermodenitrificans* in synthesizing alkylxylosides. Bioresour Technol.

[14] Matsumura S, Ando S, Toshima K, Kawada K (1998). Surface activity, antimicrobial properties and biodegradability of n-alkyl xylosides, xylobiosides, and xylotriosides. J Jpn Oil Chem Soc.

[15] Toomey J, Abboud M, Valocik R, Koster P, Burns-Kurtis C, Pillarisetti K, Danoff T, Erhardt J (2006). A comparison of the β-d-xyloside, odiparcil, to warfarin in a rat model of venous thrombosis. J Thromb Haemost.

[16] Lugemwa FN, Esko JD (1991). Estradiol β-d-xyloside, an efficient primer for heparan-sulfate biosynthesis. J Biol Chem.

[17] Kalita M, Quintero MV, Raman K, Tran VM, Kuberan B (2015). Synthesis and biomedical applications of xylosides. Methods Mol Biol.

[18] Nilsson U, Jacobsson M, Johnsson R, Mani K, Ellervik U (2009). Antiproliferative effects of peracetylated naphthoxylosides. Bioorg Med Chem Lett.

[19] Mani K, Belting M, Ellervik U, Falk N, Svensson G, Sandgren S, Cheng F, Fransson LA (2004). Tumor attenuation by 2(6-hydroxynaphthyl)-β-d-xylopyranoside requires priming of heparan sulfate and nuclear targeting of the products. Glycobiology.

[20] Nieto-Dominguez M, de Eugenio LI, Barriuso J, Prieto A, Fernandez de Toro B, Canales-Mayordomo A, Martinez MJ (2015). Novel pH-stable glycoside hydrolase family 3 β-xylosidase from talaromyces amestolkiae: an enzyme displaying regioselective transxylosylation. Appl Environ Microbiol.

[21] Box GEP, Behnken DW (1960). Some new three level designs for the study of quantitative variables. Technometrics.

[22] Salvachua D, Prieto A, Martinez AT, Martinez MJ (2013). Characterization of a novel dye-decolorizing peroxidase (DyP)-type enzyme from *Irpex lacteus* and its application in enzymatic hydrolysis of wheat straw. Appl Environ Microbiol.

[23] Yan Q, Wang L, Jiang Z, Yang S, Zhu H, Li L (2008). A xylose-tolerant β-xylosidase from *Paecilomyces thermophila*: characterization and its co-action with the endogenous xylanase. Bioresour Technol.

[24] Britton HTS, Robinson RA (1931). Universal buffer solutions and the dissociation constant of veronal. J Chem Soc.

[25] Bernabé M, Salvachúa D, Jiménez-Barbero J, Leal JA, Prieto A (2011). Structures of wall heterogalactomannans isolated from three genera of entomopathogenic fungi. Fungal Biol.

[26] Ciucanu I, Kerek F (1984). A simple and rapid method for the permethylation of carbohydrates. Carbohydr Res.

[27] Blanchard JE, Withers SG (2001). Rapid screening of the aglycone specificity of glycosidases: applications to enzymatic synthesis of oligosaccharides. Chem Biol.

[28] Batra J, Beri D, Mishra S (2014). Response surface methodology based optimization of β-glucosidase production from *Pichia pastoris*. Appl Biochem Biotechnol.

[29] Ma H, Liu WW, Chen X, Wu YJ, Yu ZL (2009). Enhanced enzymatic saccharification of rice straw by microwave pretreatment. Bioresour Technol.

[30] Marsh JW, Denis J, Wriston JC (1977). Glycosylation of *Escherichia coli*l-asparaginase. J Biol Chem.

[31] Macauley-Patrick S, Fazenda ML, McNeil B, Harvey LM (2005). Heterologous protein production using the *Pichia pastoris* expression system. Yeast.

[32] Wei W, Chen L, Zou G, Wang Q, Yan X, Zhang J, Wang C, Zhou Z (2013). N-glycosylation affects the proper folding, enzymatic characteristics and production of a fungal β-glucosidase. Biotechnol Bioeng.

[33] Imperiali B, O’Connor SE (1999). Effect of N-linked glycosylation on glycopeptide and glycoprotein structure. Curr Opin Chem Biol.

[34] Herscovics A (1999). Processing glycosidases of *Saccharomyces cerevisiae*. Biochim Biophys Acta.

[35] Deshpande N, Wilkins MR, Packer N, Nevalainen H (2008). Protein glycosylation pathways in filamentous fungi. Glycobiology.

[36] Fallico B, Lanza MC, Maccarone E, Asmundo CN, Rapisarda P (1996). Role of hydroxycinnamic acids and vinylphenols in the flavor alteration of blood orange juices. J Agric Food Chem.

[37] Vanbeneden N, Gils F, Delvaux F, Delvaux FR (2008). Formation of 4-vinyl and 4-ethyl derivatives from hydroxycinnamic acids: occurrence of volatile phenolic flavour compounds in beer and distribution of Pad1-activity among brewing yeasts. Food Chem.

[38] Ochs M, Muzard M, Plantier-Royon R, Estrine B, Remond C (2011). Enzymatic synthesis of alkyl β-d-xylosides and oligoxylosides from xylans and from hydrothermally pretreated wheat bran. Green Chem.

[39] Corre J, Lucchini JJ, Mercier GM, Cremieux A (1990). Antibacterial activity of phenethyl alcohol and resulting membrane-alterations. Res Microbiol.

[40] Saeidnia S, Yassa N, Rezaeipoor R, Shafiee A, Gohari A, Kamalinejad M, Goodarzy S (2009). Immunosuppressive principles from *Achillea talagonica*, an endemic species of Iran. DARU J Pharm Sci.

[41] Shinoyama H, Kamiyama Y, Yasui T (1988). Enzymatic synthesis of alkyl β-xylosides from xylobiose by application of the transxylosyl reaction of *Aspergillus niger* β-xylosidase. Agric Biol Chem.

[42] Jacobsson M, Ellervik U, Belting M, Mani K (2006). Selective antiproliferative activity of hydroxynaphthyl-β-d-xylosides. J Med Chem.

[43] Siegbahn A, Aili U, Ochocinska A, Olofsson M, Ronnols J, Mani K, Widmalm G, Ellervik U (2011). Synthesis, conformation and biology of naphthoxylosides. Bioorg Med Chem.

[44] Johnsson R, Mani K, Ellervik U (2007). Synthesis and biology of bis-xylosylated dihydroxynaphthalenes. Bioorg Med Chem.

[45] Choengpanya K, Arthornthurasuk S, Wattana-amorn P, Huang WT, Plengmuankhae W, Li YK, Kongsaeree PT (2015). Cloning, expression and characterization of β-xylosidase from *Aspergillus niger* ASKU28. Protein Expr Purif.

[46] Xia W, Shi P, Xu X, Qian L, Cui Y, Xia M, Yao B (2015). High level expression of a novel family 3 neutral β-xylosidase from *Humicola insolens* Y1 with high tolerance to d-xylose. PLoS One.

[47] Li H, Wu J, Jiang F, Xue Y, Liu J, Gan L, Ali N, Long M (2015). Functional expression and synergistic cooperation of xylan-degrading enzymes from *Hypocrea orientalis* and *Aspergillus niger*. J Chem Technol Biotechnol.

[48] Kirikyali N, Connerton IF (2014). Heterologous expression and kinetic characterisation of *Neurospora crassa* β-xylosidase in *Pichia pastoris*. Enzym Microb Technol..

[49] Vasu P, Bauer S, Savary BJ (2012). Cloning and expression of hemicellulases from *Aspergillus nidulans* in *Pichia pastoris*. Methods Mol Biol.

[50] Ohta K, Fujimoto H, Fujii S, Wakiyama M (2010). Cell-associated β-xylosidase from *Aureobasidium pullulans* ATCC 20524: purification, properties, and characterization of the encoding gene. J Biosci Bioeng.

[51] Wakiyama M, Yoshihara K, Hayashi S, Ohta K (2008). Purification and properties of an extracellular β-xylosidase from *Aspergillus japonicus* and sequence analysis of the encoding gene. J Biosci Bioeng.

