# Supplementary material for: Enzymatic fine-tuning for 2-(6-hydroxynaphthyl) β-d-xylopyranoside synthesis catalyzed by the recombinant β-xylosidase BxTW1 from Talaromyces amestolkiae
Source: Microb Cell Fact. 2016 Oct 4;15:171. doi: 10.1186/s12934-016-0568-6 (PMC5050587; doi:10.1186/s12934-016-0568-6)
Supplement: Supplementary file 1 — 10.1186/s12934-016-0568-6 Xylose and xylobiose as transxylosylation acceptors. Transxylosylation reactions catalyzed by rBxTW1 using xylose or xylobiose as acceptors. The analysis by TLC of the samples is displayed in order to demonstrate the synthesis of the transxylosylation products. [file 12934_2016_568_MOESM1_ESM.docx]

**ADDITIONAL FILE 1**

**Xylose and xylobiose as transxylosylation acceptors**. The capacity of the native BxTW1 for the transxylosylation of xylose and xylobiose was fully proven in a previous work [1]. To confirm that rBxTW1 kept the same properties, additional experiments were carried out with the recombinant enzyme. For xylobiose analyses the reaction mix contained 0.5 mU/mL rBxTW1, 50 mM xylobiose as donor and acceptor, 0.1% BSA, 50 mM sodium formate buffer (pH3). Reactions for testing the xylose role as acceptor were prepared by adding 5 U/mL rBxTW1, 20 mM *p*NPX as donor, 150 mM xylose as acceptor, 0.1% BSA, 50 mM sodium formate buffer (pH3). In both cases reaction mixtures were incubated at 50 °C and 1,200 rpm for 10 min and stopped by heating at 100 °C for 5 min. The same reaction mixes without enzyme were added as controls and a solution of X1-X4, each one at a final concentration of 4 g/L, was prepared as standard in order to follow the synthesis of the new products by TLC.

Tin layer chromatographies (TLCs) were carried out in 1-butanol/acetic acid/water 2:1:1 (v/v). Detection was performed by immerse the membrane in a methanol/sulfuric acid 95:5 (v/v) solution and heating at 110 °C for 5 min [2]. When xylobiose mixtures were analyzed, two new spots appeared besides the disaccharide (Fig. S1A), one with the migration distance of xylose and the other one corresponding to xylotriose. While xylose can be a product of normal hydrolysis or of transxylosylation (since one xylose moiety is always released with the first nucleophilic attack), the presence of xylotriose clearly indicated transxylosylation with xylobiose as acceptor. Regarding the reaction with *p*NPX and xylose (Fig. S1B), the emergence of a new spot at the migration distance of xylobiose confirmed the role of xylose as acceptor of transxylosylation. *p*NPX (the donor) seemed to be totally consumed, but the detection method prevent the vision of the released *p*NP. Finally, the intensity of the xylose spot was coherent with its large excess.

Fig S1. TLCs from transxylosylation reactions catalyzed by rBxTW1 using xylobiose (A) and xylose (B) as acceptors. Both assays display a marker of X1-X4, the reaction pattern and a control without enzyme.

**REFERENCES**

1. Nieto-Dominguez M, de Eugenio LI, Barriuso J, Prieto A, Fernandez de Toro B, Canales-Mayordomo A, Martinez MJ. Novel pH-stable glycoside hydrolase family 3 β-xylosidase from *Talaromyces amestolkiae*: an enzyme displaying regioselective transxylosylation. Appl Environ Microbiol. 2015;81:6380-92.

2. Gil-Muñoz J. Estudio de las β-glucosidasas del complejo celulolítico de *Talaromyces amestolkiae*: Caracterización y aplicaciones biotecnológicas. Thesis, Universidad Complutense, Madrid. 2015
